# Supplementary material for: The price penalty for red meat substitutes in popular dishes and the diversity in substitution
Source: PLoS One. 2021 Jun 17;16(6):e0252675. doi: 10.1371/journal.pone.0252675 (PMC8211254; doi:10.1371/journal.pone.0252675)
Supplement: Attachment — Submitted filename: Respone to Reviewers.docx [file pone.0252675.s001.docx]

Dear Damian Adams,

Thanks for organizing the constructive review process. For the formal requirements I used a template that I thought applies to the registered reports in PLOS ONE. After checking the link with the guidelines, I made manuel changes. Please let me know, if I overlooked a requirement. The comment of sharing the research data in a public repositorium is probably not applicable to the registered report stage. However, once the data is collected, I can update you so that a link can be added to the registered report, if you like.

Dear Reviewers,

I appreciate your time and interest to review the manuscript. You have triggered substantial improvements. The track-change version looks a bit messy because I restructured the sections a bit. The main agenda of the manuscript has not changed but many helpful additions make the presentation of the study more comprehensible. I tried to respond to each comment one by one. The response is a bit longer if I did not fully comply with your suggestion or if you raised a question.

6. Review Comments to the Author

**Reviewer #1**: The paper was presented as Registered Report Protocol and discusses the research design, methodology and its implementation for estimating the price penalty (defined as willingness to pay) for replacing red meat dishes. In particular, the element that seems to lead the author to investigate the willingness to pay for the replacement of the Western diet is the high environmental impact and unhealthiness of a diet heavily skewed towards red meat consumption. In this regard, in the introduction, the author underlines the lack of evidence in the scientific literature regarding the trade-off that consumers face when asked to change their eating habits.
The study is therefore part of a series of sustainable consumption models, and seeks to highlight a critical aspect that has received far too little attention from the scientific community seeking to propose new models of sustainable production and consumption: the consumer and his habits.
The author assumes that consumers are willing to substitute the meat dishes they normally consume in return for a price penalty (lower willingness to pay) and this is fundamentally acceptable given that the target of the research also aims to highlight the willingness to pay on the part of frequent red meat consumers, therefore with very well rooted eating habits.
My main concern is how the environmental aspect was considered in the study. In fact, the author states that "The research objectives are complemented by exploratory research on the relationship of consumer characteristics with a preference for a substitution option and a calculation of what CO2 taxing level would flip the preference structure if any". The environmental aspect, which was heavily emphasised in the introduction of the work, takes a back seat and becomes a complementary element. This also emerges in the paragraph on the definition of attributes, where the author states that the choice set includes only three attributes: dish, price and waiting time.
Certainly the dish must be one of the attributes, just as the price is essential in determining willingness to pay. However, the choice of the third attribute, waiting time, even if as the author states "represents the value of time which matters particularly in lunch settings and hints at a convenience factor of interest to all kinds of food interventions" should be evaluated more thoroughly.
In my opinion, given that the whole study is designed to assess the link between sustainable consumption patterns and consumer willingness to adopt them, one of the three attributes (if the author does not consider it appropriate to add a fourth) should clearly emphasise the environmental qualities of the substitute dishes, otherwise the consumer's choice of preferred combination of attributes is completely disconnected from the environmental and health characteristics on the basis of which the need to replace the use of red meat in dishes arises.

The point is very well taken. My perspective is that consumer choices depend primarily on taste preferences and availability, while CO2-footprints are not strong behavioral cues. Several consumer studies in real purchase scenarios have failed to significantly influence food habits via CO2 labelling, e.g. (Kortelainen, Raychaudhuri, and Roussillon 2016; Gert et al. 2013). Since the Friday for Future Movement, the studies might be a bit outdated. I agree that the set up and motivation of the study calls for a consideration of CO2-footprints to analyze how much such information might be able to contribute to closing the price penalty gap. I chose to include information about CO2-emissions in the choice design. The information is presented in a simple manner, because there is evidence that exact CO2-values are too abstract for consumers, e.g. (Meyerding, Schaffmann, and Lehberger 2019). I sincerely hope that the change of a dish’s CO2 footprint between choice sets is believable and look forward to the results.
This aspect, highlighted by the author in Table 1, should be clearly explained to the consumer. In the paragraph on the choice procedure, it is stated that the dishes are explained neutral, however it should be pointed out which aspect of the dishes was explained: only the main ingredients or also the related CF data? I know this may seem like a quibble but it isn't because it would place the study clearly and unequivocally in the context in which the author stated he wanted to place the study from the very first lines of the introduction.

Yes, neutral introduction is not a good description of what I had in mind. The main ingredients of the dishes are named but the dishes are not introduced. The research question are best answered and generalized if consumer beliefs concerning each dish are not subject to the research design. The experiment is intended to capture the current status quo of the market, not to evaluate the effect of information. This may also help to avoid social desirability bias. I rephrased the paragraph.
In this same vein, the author should better explain how he wants to relate the results of the IACBC to those of the Carbon Footprint calculation and thus the carbon tax. How might these relationships affect the preference structure and why?

The idea is to calculate the price on CO2-e necessary to compensate for the price penalty of the substitutes. This requires the information in Table 2 on the CO2-e difference and the choice set results on the WTP, i.e. the price penalty for the red meat dish. Possible implications are that the CO2-tax level to compensate the price penalty is (not) justifiable. As CO2 taxes are increasingly discussed, the findings here show whether such a tax would affect the willingness to substitute. Now, I briefly explain this in the section “research objective”.

The paragraphs on Sampling procedure, Choice procedure, IACBC and data analysis are well structured, clear and provide the reader with all the necessary information to understand how the methodology under study will be implemented.
As a suggestion for the improvement of the study I suggest the author to better investigate the scientific literature on the application of LCA methodology to different diets, because the studies cited are a bit 'dated and much has been published in this direction by the scientific community.

I appreciate the comments. The restructuring of the manuscript has primarily led to an improvement concerning the scientific literature on consumer studies as the main contribution of this research. I also strengthened the LCA motivation in the introduction.

Reviewer #2: The paper discusses consumer preferences on the substitution of red meat used in the preparation of different food receipts. Despite the interest of the topic, the paper suffers from severe limitations because it does not represent a complete analysis and is lacking from results.

1 title The authors should rethink about the title; maybe better not to mention the examples (red-meat dishes) and just highlight their focus on red meat. However, this is only a personal idea (that does not mean they should do it).

I wanted to add something tangible but I am also happier with the new version of the title
2 Introduction Globally, the introduction should be restructured and rephrased.

The upcoming comments have helped to improve the manuscript with respect to the Introduction. Generally, the introduction is restructured to better illustrate the literature gap and the consumer research on dish substitution. I added literature and better explain the substitution categories within the introduction. The additions to the introduction are build on the former section theoretical background with improvements here and there.
3 line 25 the first sentence can be better explained and referenced; alternatively, it should be better to remove it.

Ok. Now, I start with referenced information on environmental problem of red meat consumption.
4 lines 25-33 better to rephrase, highlighting more who says what (references).

I think it is clearer now. Generally, I meant to keep it brief, as many studies find various ways to describe the environmental and health issues of red meat. As this is not my research contribution, I try to avoid being repetitive. If you feel more information is demanded, let me know.
5 lines 25-42 From the beginning, the authors should clearly state and justify their focus on Germany.

I changed the introduction to a European/western diet context. In general, the described and analyzed problem of the price penalty for substitutes should not be understood as limited to the European context. Unfortunately, a multi-cultural study is not feasible at this point.
6 lines 49-50 "Consumer choices, however, occur on a dish or ingredient level, since the majority of consumers is not committed to a specific diet." Are the authors sure of what they mention? Can they provide a reference for this statement? Honestly, I am not sure this is true (e.g. think about the Mediterranean diet). This cannot be generalised. The authors should better specify it with a reference.

Good point. I changed the manuscript to be more clear about what I mean here. In general, we need transition strategies on diet and dish levels, which for example Boer and Aiking’s (s.b.) review emphasizes.
7 line 56 "Additionally, in line with a review by Boer and Aiking (3), this study accounts for the multitude of options to substitute". At the end of this sentence, I would ask: "to do what?". I mean that the authors here introduce this sentence to only partially explain what they will do (within the research). I think the aim should be better and clearly stated.

I included the consumer research on substitution options and preferences in the introduction. This should help the reader to understand the type of substitution asked of consumers.
8 lines 56-60 Where do these options come from? A previous focus group? An existing study? It should be explained to justify them.

I abstracted them from the consumer research studies but the review by Boer and Aiking has previously described the substitution categories in a very similar fashion.
9 line 60 Here, the authors mention Spaghetti Bolognese for the first time, without explaining why they mention this. It is not clear for the reader why.

I removed the concrete dishes from the introduction, as this is more of a methodological choice.
10 lines 60-63 The same of comment n. 8 (please, see above)

The selection of the options is now briefly described in separate section “Three popular meat dishes”
11 line 67 Please, remove "I"

Sure
12 lines 67-68 This should proceed with what stated in lines 56-63.

Your comments have triggered a restructuring that I feel has significantly improved the introduction
13 Theoretical background: I wonder why these four substitutes are being applied to the German sample. Are there any similar studies, before this, dealing with a similar topic on this sample, showing the existing of a knowledge gap that this study could/might fill? I have the idea that the choice of the sample is not justified at all. Moreover, the paragraph globally should be revised, improved both with literature and own description of what the authors are presenting.

Again, the comments have triggered a restructuring of the manuscript. I added literature and better explain the substitution categories within the introduction. The additions to the introduction are build on the former section theoretical background with improvement here and there. Concerning the choice of the sample, the research objective addresses the challenge for consumers in western countries to substitute red meat. Therefore, the main objective of the sample is to provide a nearly representative consumer sample of a western country. Ideally, a multi-country analysis would be feasible. Due to budgetary constraints and expertise, I opt for a quoted consumer sample in Germany.
14 lines 77-79 Please, improve this part with a more in-depth literature analysis on consumer behaviour on substitution preferences (see your statement: line 54). This is important before starting to describe the four types of substitutes you consider in your study.

I significantly improved the illustration of consumer behavior studies concerning the substitution option. I added more findings. As previously explained this part is now integrated in the introduction to explain what we know already and to explain the research gap that the studies have not addressed.
15 line 111 Please, get rid of "I". Moreover, what is the role of "whether" in that sentence?

Adjusted
16 line 112 I think the authors mean the four substitutes ("for each dish"). As the authors previously stated, CO2 footprints are based on estimations by IFEU-institute. So, what have the authors done here? Just compared the CO2 footprints of Spaghetti Bolognese, beef topping for 2 buns with butter, and beef meatballs with rice and peas with the relative 4 substitutes? Or have they previously measured the CO2 footprints for the substitutes, before comparing these with that retrieved from IFEU. This is not clear to me.

The revised sentence should be clearer. The CO2 footprints for the dishes at hand are based on IFEU institute data. The contribution of this manuscript will be the online experiment on consumer preferences concerning substitution options with lower CO2-footprints. Please note, the experiment has not been implemented at this point.
17 Research objective In this paragraph, the authors state their hypothesis but these are not supported by any relevant previous reference in the literature. It seems to me that such a kind of analysis is a bit self-referential, probably it does not fit the scope of a scientific publication at all.

The research is not self-referential. I think no Author occurs more than twice, maybe not even more than once in the reference list. As for the research objective, I understand this is not a typical way of presenting it. I added literature to make a stronger case on why I assume a price penalty for all substitution options, which also follows from the introduction. The hypothesis on an ordinal preference structure between substitution options is still not backed with empirical data. The reason is the described research gap of how consumer research has hardly addressed multiple substitution categories in a single study framework. I hope the revised Introduction and Research objective feel acceptable.
18 lines 122-123 "Empirical evidence on the WTP for substitution pathways is scarce". I suggest the authors use this statement also within the previous paragraph, to help them explain why they are doing this kind of research.

Indeed, it would be better to lead with that. Generally, I restructured the Introduction and Research Objective
19 line 124 Please, get rid of "I".

Instead of switching to passiv mode, I changed all “I” to “we”. Please let me know, if you prefer the passive mode.

20 Study design: The last part of this paragraph should be stated previously, to justify the research (e.g. describing possible implication, although very briefly).

Indeed, this is still research justification and better fits with the introduction. I adjusted that.
21 Variables: In this paragraph, the authors should explain all the variables they mention to consider in their research (explanation is provided only for out-of-home consumption habits and consumption habits). Moreover, a table would be used to report all the items and scales. Moreover, the scale mentioned in line 159 should be the opposite (or did the authors reverse the scale? In this case, it should be explained). This paragraph does not provide enough information and should be deeply improved.

I think, it is a wise suggestion to start a table for items and scales planned. Now, Table 3 summarizes the concepts and scales. I started the programming of the experiment, so I would also be able to present the XML-code of the questionnaire if desired. Feel free to suggest concepts that may strengthen the research. Please note what is probably known, the literature for the scales can appear outdated because consumer research has started to develop useful concepts for consumer characteristics a long time ago. These concepts can still hold value to the analysis at hand.
22 line 148 Please, get rid of "I".

Adjusted
23 lines 185-200 The authors should clearly state why they chose those three attributes (e.g. inspired by any relevant paper?). Moreover, both the attributes and their levels should be included in a table: this could make it easier for the reader to understand.

Good point, I have added Table 5, so readers can better grasp the attributes and levels in the choice sets. The selection of the attributes is argued briefly within the manuscript. I strongly believe, the combination of attributes has not previously been applied in consumer research.
24 Results and conclusions are missing in the paper.

This is the registered report format
